# Maternal *H. pylori* is associated with differential fecal microbiota in infants born by vaginal delivery

**DOI:** 10.1038/s41598-020-64296-7

**Published:** 2020-04-29

**Authors:** Caroll D. Hernandez, Hakdong Shin, Paula A. Troncoso, Macarena H. Vera, Andrea A. Villagran, Selena M. Rodriguez-Rivera, Marlene A. Ortiz, Carolina A. Serrano, Arturo Borzutzky, Maria Gloria Dominguez-Bello, Paul R. Harris

**Affiliations:** 1 Department of Pediatric Gastroenterology and Nutrition, Pontificia Universidad Católica de Chile School of Medicine, Santiago, Chile; 2 Department of Food Science and Biotechnology, College of Life Science, Sejong University, Seoul, South Korea; 3 Wayne State University School of Medicine, Detroit, Michigan USA; 4 Department of Pediatric Infectious Diseases and Immunology, Pontificia Universidad Católica de Chile School of Medicine, Santiago, Chile; 5 Millennium Institute on Immunology and Immunotherapy, Pontificia Universidad Católica de Chile School of Medicine, Santiago, Chile; 6 Department of Biochemistry and Microbiology, and Department of Anthropology, Rutgers University, New Brunswick, USA; 7 Present Address: Department of Pediatrics, School of Medicine, Universidad de La Frontera, Temuco, Chile

**Keywords:** Microbiology techniques, DNA sequencing, Microbiome, Paediatric research

## Abstract

*Helicobacter pylori* colonization may affect the mucosal immune system through modification of microbiota composition and their interactions with the host. We hypothesized that maternal *H. pylori* status affects the maternal intestinal microbiota of both mother and newborn. In this study, we determine the structure of the fecal microbiota in mothers and neonates according to maternal *H. pylori* status and delivery mode. We included 22 mothers and *H. pylori* infection was determined by fecal antigen test. Eleven mothers (50%) were *H. pylori*-positive (7 delivering vaginally and 4 by C-section), and 11 were negative (6 delivering vaginally and 5 by C-section). Stool samples were obtained from mothers and infants and the fecal DNA was sequenced. The fecal microbiota from mothers and their babies differed by the maternal *H. pylori* status, only in vaginal birth, not in C-section delivery. All 22 infants tested negative for fecal *H. pylori* at 15 days of age, but those born vaginally –and not those by C-section- showed differences in the infant microbiota by maternal *H. pylori* status (PERMANOVA, p = 0.01), with higher abundance of *Enterobacteriaceae* and *Veillonella*, in those born to *H. pylori*-positive mothers. In conclusion, the structure of the infant fecal microbiota is affected by the maternal *H. pylori* status only in infants born vaginally, suggesting that the effect could be mediated by labor and birth exposures.

## Introduction

*Helicobacter pylori*, a gastric pathobiont, dominates in the gastric microbiota^1^. Since the role of microbiota in health and disease has become more apparent with advances in sequencing techniques, it is now possible to underst and the interplay between *H. pylori* and the gastrointestinal microbiota, especially early in life, when the composition of the microbiota is established and then matures during the first 3 years of life^2^.

While *H pylori* is associated with significant morbidity during adult life, the apparent - still controversial- beneficial role in infants, might be related to a developmental effect. Indeed, *H. pylori* has been suggested to prime a “healthy” mucosal immune response, by upregulating the T regulatory (Treg) response leading to protection against allergies and asthma^3–8^. *H. pylori* induction of Tregs, particularly in children, may be accountable for the decrease of Th2 responses, which are elevated in allergies. This effect, directly associated to *H. pylori* carriage, might be mediated by the microbiota and its interplay with the host. Since *H. pylori* infection alters gastric physiology (i.e.: changes in pH) and gastric microbiota, the more distal intestinal microbiota might also be affected, as it has been shown in Mongolian gerbils, mice and humans^9–12^.

A birth cohort study has shown the early stool detection of *H. pylori* during the first year of life, reaching 20% of the children by age 5 years^13^. We have demonstrated that the presence of *H. pylori* in children is associated with significant differences in the gastric microbiota, with higher diversity, reduced abundance of Firmicutes and increased abundance of non-*Helicobacter*-Proteobacteria, and higher gastric mucosal FOXP3 expression, IL-10 and TGF-β than non-infected subjects^1^. This suggests that early life *H. pylori* colonization contributes to immune tolerogenic responses^5^. However, whether at the beginning of extrauterine life, before the window of *H. pylori* colonization, developmental immune responses are also modulated by the maternal *H. pylori* status, mediated by microbiota-related factors, is unknown. Since the maternal microbiota shapes maternal immunity, and is the source for the offspring microbiota, we hypothesize that maternal *H. pylori* status modulates the fecal microbiota structure in infants. Here we determined the structure of the intestinal microbiota in mothers with and without *H. pylori *infection, and their newborns.

## Results

### General characteristics of the mothers

Twenty-two consecutive mothers and their newborns were recruited. Eleven mothers were *H. pylori*-positive (50%), of which 7 (63.6%) mothers underwent vaginal delivery and 4 (36.4%) underwent C-section. The other 11 mothers were *H. pylori*-negative, of which 6 (54.5%) of them underwent vaginal delivery and 5 (45.5%) mothers underwent C-section. The mean maternal age was 30.9 ± 4.8 for *H. pylori*-positive mothers and 29.3 ± 6.3 for *H. pylori*-negative mothers. No differences were found for type of delivery between infected and non-infected mothers (p = 0.7) or for maternal age (p = 0.5) (Table 1).

**Table 1:** Characteristics of the subjects according to maternal *H. pylori* infection.

	Maternal *H. pylori* (+)	Maternal *H. pylori* (−)	P value
Total mother-newborn dyad	n = 11	n = 11	
Maternal age, years ± SD	30.9 ± 4.8	29.3 ± 6.3	0.5
Delivery route, n (%)			
Vaginal	7 (63.6)	6 (54.5)	0.7
C-section	4 (36.4)	5 (45.5)	
Maternal nutritional status, n (%)			
Underweight, BMI < 18.5	1 (9.1)	1 (9.1)	0.5
Normal, BMI: 18.5–24.9	1 (9.1)	4 (36.4)	
Overweight, BMI: 25–29.9	6 (54.5)	4 (36.4)	
Obese, BMI >30	1 (9.1)	2 (18.1)	
Group B streptococcus (GBS) infection, n (%)	1 (9.1)	1 (9.1)	1
Maternal use of antibiotics in the last 3 months, n (%)	0 (0)	2 (18.2)	ND
Newborn gender, n (%)			
Female	4 (36.4)	8 (72.7)	0.2
Male	7 (63.6)	3 (27.3)	
Newborn weight, kg ± SD	3.4 ± 0.1	3.5 ± 0.1	0.5
Newborn height, cm ± SD	50 ± 0.5	50 ± 0.4	0.9
Feeding, n (%)			
Exclusively breastfeeding	8 (72.7)	8 (72.7)	1
Predominantly breastfeeding	3 (27.3)	3 (27.3)	
Exclusively formula-fed	0 (0)	0 (0)	
Inhabitants per household, n ± SD	3.9 ± 0.3	4.4 ± 0.7	0.5
Socioeconomic status*, n (%)			
High	4 (36.4)	3 (27.3)	0.6
Middle	7 (63.6)	8 (72.7)	

^*^Socioeconomic status according to ESOMAR score. ND: no-determinate statistically.

No differences were found in maternal nutritional status between the groups (p = 0.5). For *H. pylori*-positive mothers 9.1% were normal, 9.1% were underweight, 54.5% were overweight and 9.1% were obese. For non-infected mothers 36.4% were normal, 9.1% were underweight, 36.4% were overweight and 18.1% were obese.(Table 1).

At delivery only 2 mothers had infection by group B streptococcus (GBS), one of them was *H. pylori*-positive mother (who delivered vaginally and received GBS antibiotic treatment). In the last 3 months of pregnancy, 2 *H. pylori*-negative mothers used antibiotics and one of them also had GBS infection (C-section delivery) (Table 1).

### General characteristics of the newborn

In total, 12 newborns were female (4 from *H. pylori*-positive mothers and 8 from *H. pylori*-negative mothers) and 10 were male (7 from *H. pylori*-positive mothers and 3 from *H. pylori*-negative mothers), without significant differences (p = 0.2). The mean birth weight was 3.4 ± 0.1 kg for newborns from infected mothers and 3.5 ± 0.1 kg for newborns from non-infected mothers. The mean height was 50 ± 0.5 cm and 50 ± 0.4 cm for newborns from infected and non-infected mothers respectively. No differences were found in weight and height between children from infected and non-infected mothers (p = 0.5 and p = 0.9, respectively) (Table 1).

At 15 days of age, no newborns were *H. pylori* positive based on fecal antigens, and 16 of the 22 were exclusively breastfed (8 in each group), 6 were predominantly breastfed (3 in each group) and none were exclusively formula fed. The inhabitants per household were 3.9 ± 0.3 for the *H. pylori*- positive group and 4.4 ± 0.7 for the *H. pylori*- negative group. There were no differences in feeding patterns, socioeconomic level, or number of family members living in the same house between newborns from infected and non-infected mothers (Table 1).

### Maternal and infant fecal microbiota structure

We obtained 1,075,570 sequence reads in total, 539,608 reads from 22 newborn fecal samples and 535,962 reads from 22 mother fecal samples (paired-end, >Q19), binned into 2,845 Operating Taxonomic Units (OTUs) (Table S1).

### Structure of fecal microbiota samples from mothers according to *H. pylori* infection status and mode of delivery

**Figure 1:**
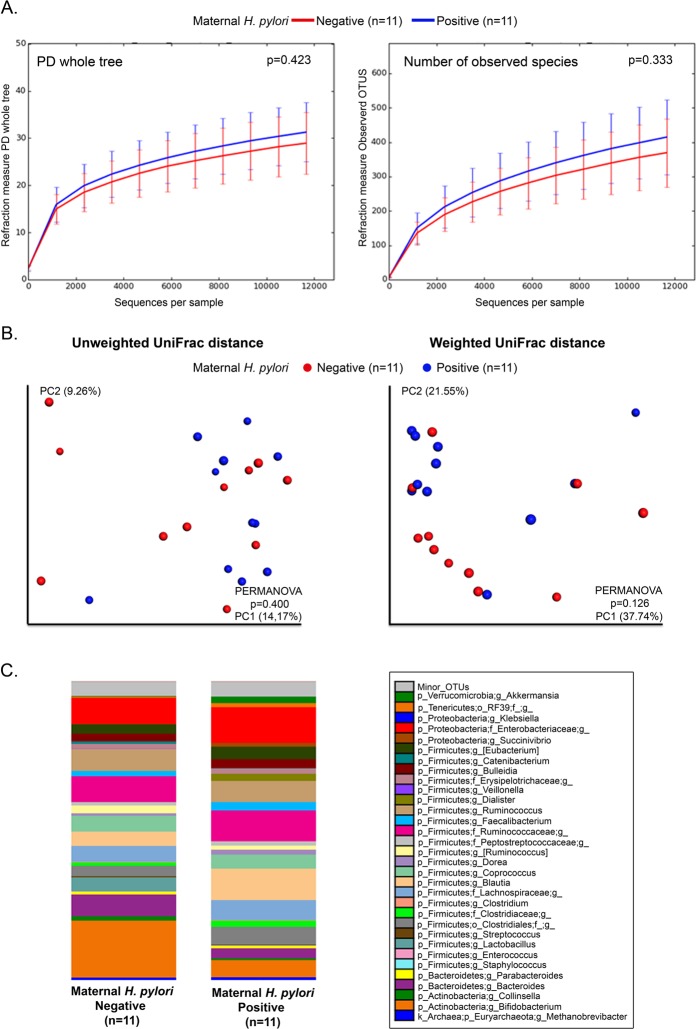
Bacterial communities of mother’s feces by *H. pylori* status. (**A**) Rarefaction plots of bacterial communities using PD whole tree matrix (Left) and number of observed species (Right). The non-parametric P values were calculated using 10,000 Monte Carlo permutation. **(B)** PCoA plot of bacterial communities using unweighted (Left) and weighted (Right) UniFrac distances. PERMANOVA was used to test dissimilarity. **(C)** Bacterial taxa plots. Each taxonomy (>1% of average relative abundance in any groups) is indicated by a different color at the genus level. LDA Effect Size (>3.0-fold) was used to detect overrepresented taxa.

**Figure 2:**
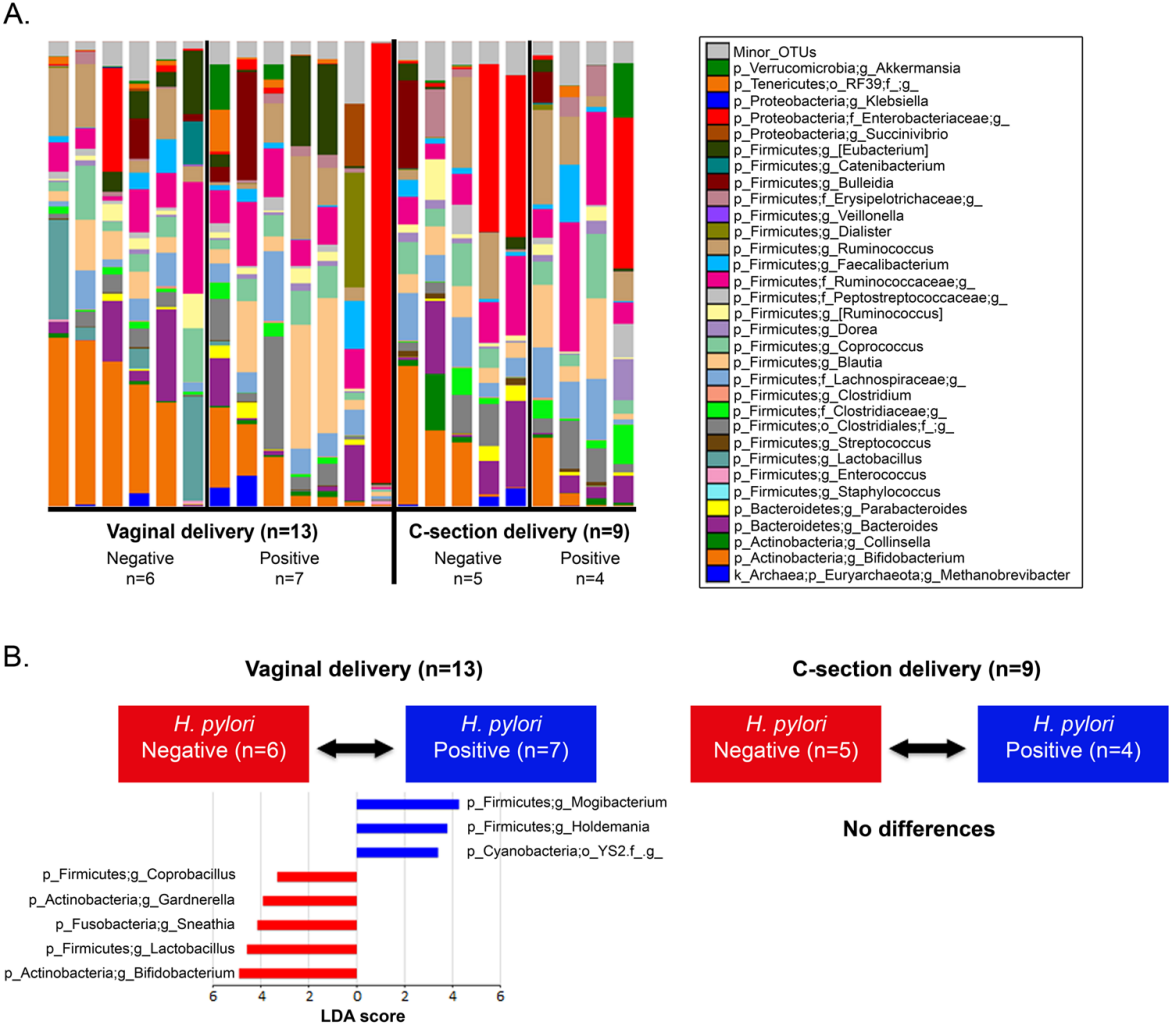
Bacterial taxa discriminant in intestinal microbiota from mothers. (**A**) Bacterial taxa plots in intestinal microbiota from each mother by *H. pylori* status according to mode of delivery. Each taxonomy is indicated by a different color at the genus level. **(B)** Histogram of overrepresented taxa in maternal faces by *H. pylori* status (LEfSe analysis between two groups, LDA > 3.0; approx. p < 0.001).

No differences were detected in maternal fecal alpha (Fig. 1A), or beta diversity (Fig. 1B) by *H. pylori* status (Fig. 1C), but when accounting for delivery mode, mothers that delivering vaginally who were *H. pylori*-positive had lower abundance of *Lactobacillus* and *Bifidobacterium* (power analysis total sample size 70 and 76, respectively) (LDA > 3.0; >1% OTUS in any groups). No significant differences were found in mothers undergoing C-section (Fig. 2A,B). Thus, vaginal delivery but not C-section show differences in the maternal microbiota by *H. pylori* status.

### Microbiota diversity in infant fecal samples according to the maternal *H. pylori* status and mode of delivery

**Figure 3:**
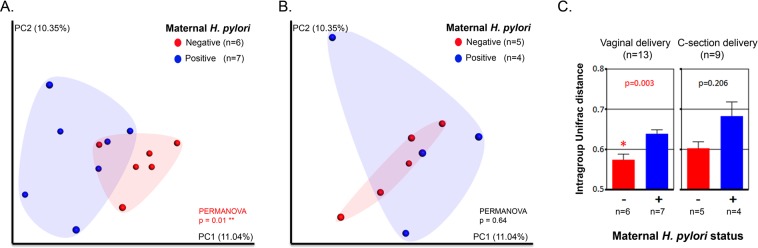
Bacterial beta diversity in infant feces by maternal *H. pylori* status and delivery mode. PCoA plot of bacterial communities using unweighted UniFrac distances in vaginally (n = 13) **(A)** or C-section (n = 9) **(B)** born infants. Unweighted UniFrac intragroup distances **(C)**. PERMANOVA was used to test dissimilarity. The non-parametric P values were calculated using 10,000 Monte Carlo permutations.

**Figure 4:**
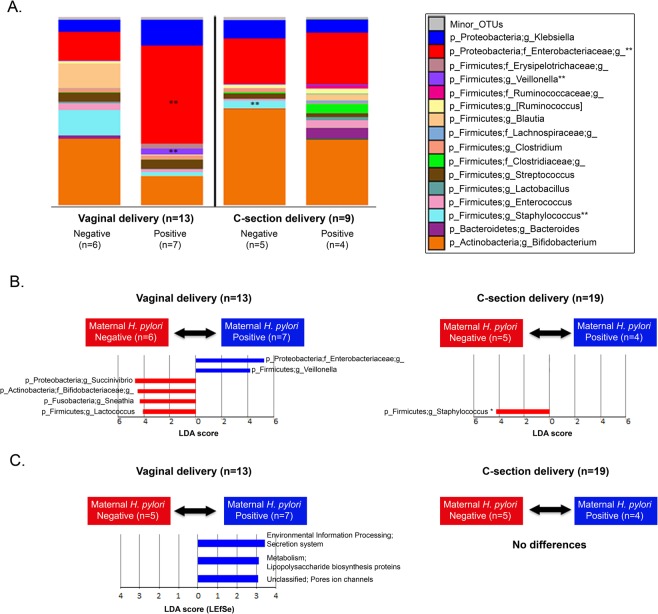
Bacterial taxa and predictive functional profiling comparisons in intestinal microbiota from infants by maternal *H. pylori* status according to mode of delivery. (**A**) Bacterial taxa plots. Each taxonomy (>1% of average relative abundance in any groups) is indicated by a different color at the genus level. (**) indicates overrepresented major taxa. (**B**) Histogram of overrepresented taxa in each group (LEfSe analysis between two groups, LDA > 3.0; approx. p < 0.001). (**C**) Histogram of overrepresented functional profiles in each group. Overrepresented functional routes in predicted metagenomes were detected using LDA Effect Size test (LEfSe analysis between two groups, LDA > 3.0; approx. p < 0.001).

**Figure 5:**
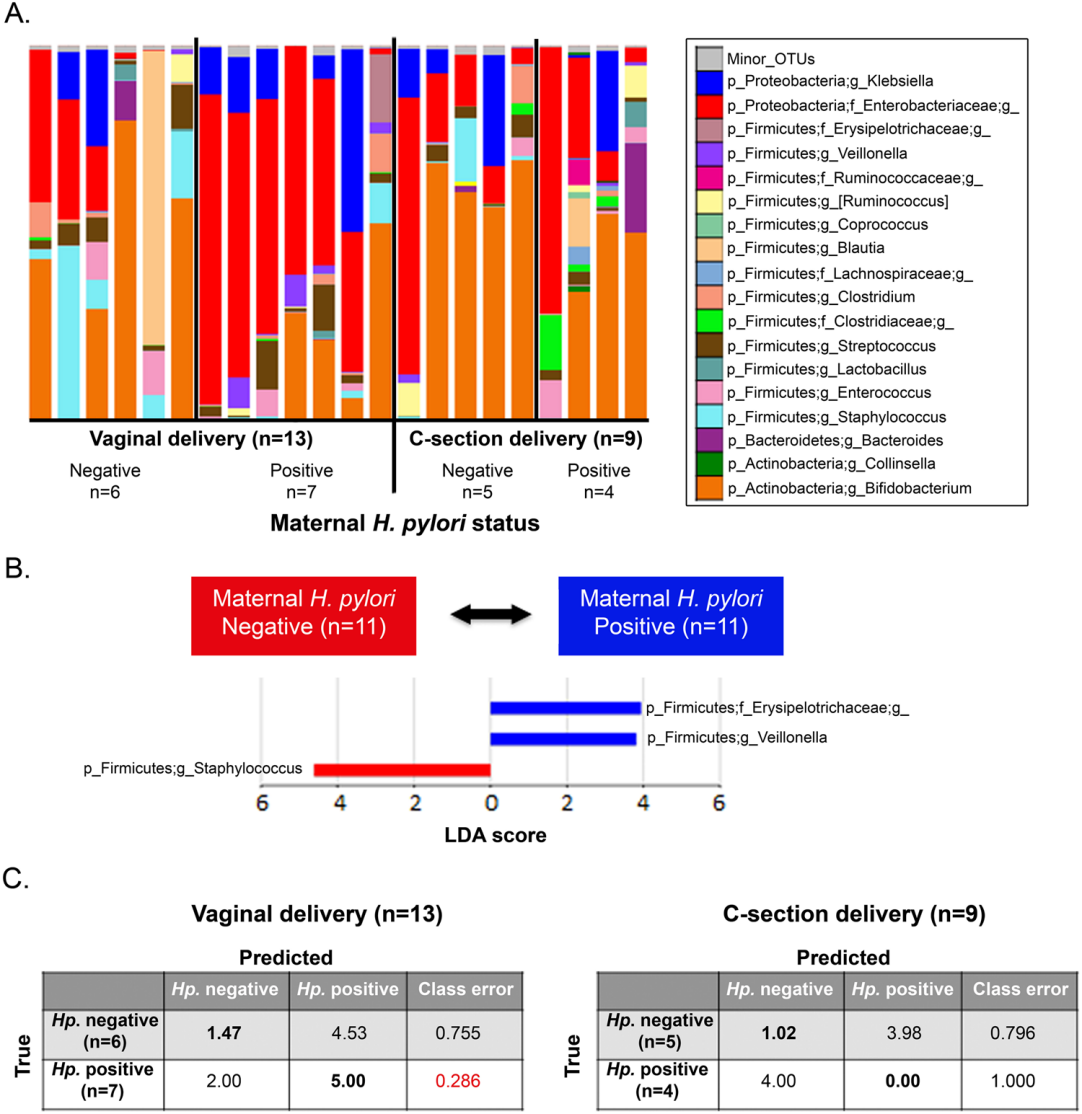
Individual taxa plots and bacterial taxa discriminant in infant feces by maternal *H. pylori* status. (**A**) Bacterial taxa plots in intestinal microbiota from each infant by maternal *H. pylori* status according to mode of delivery. Each taxonomy is indicated by a different color at the genus level. **(B)** Histogram of overrepresented taxa in infant faces by maternal *H. pylori* status (LEfSe analysis between two groups, LDA > 3.0; approx. p < 0.001). **(C)** Random Forest prediction of maternal *H. pylori* status based on her child’s fecal microbiota structure. The confusion matrix of infant fecal samples is based on a Random Forest classifier (loop method) over 100 rarefactions of the full OTU tables.

Bacterial community structures in infant fecal samples segregated by maternal *H. pylori* status only in infants born vaginally (Unweighted UniFrac distance; PERMANOVA, p = 0.01; Fig. 3A,B and Fig. S1). Vaginally delivered infants born to *H. pylori*-negative mothers had significantly reduced variability in their fecal bacterial structure in relation to vaginally born infants from *H pylori*-positive mothers (non-parametric t-test on intragroup distances; p = 0.003). This maternal status dependent-difference was not significant in C-section delivered infants (p = 0.206; with power analysis total sample size = 38) (Fig. 3C). There were no significant differences in infant fecal alpha diversity by maternal *H. pylori* status (Fig. S2), but there were differences in bacterial composition. Vaginal newborns to *H pylori*-positive mothers showed-in relation to those born to *H pylori*-negative mothers- higher abundance of *Enterobacteriaceae* and *Veillonella* (power analysis, total sample size respectively = 2,708 and >10,000) and lower *Bifidobacteriaceae*, *Succinivibrio, Sneathia*, and *Lactococcus* (LDA > 3.0; >1% OTUs in any groups) (Fig. 4A,B). To support our results, we analyzed the relative abundances of *Enterobacteriaceae* and *Veillonella*based on SILVA and EzBioCloud16S databases (Fig. S3). In opposition, infants born to *H. pylori*-negative mothers, had higher representation of fecal *Staphylococcus* and lower *Erysipelotrichaceae* and *Veillonella* (LDA > 3.0; Fig. 5A,B). In these 15 day old neonates, microbiota differences by maternal status were less pronounced than by delivery mode.

Using Random Forest prediction (with loop method) of maternal *H. pylori* status based on her infant’s fecal microbiota structure, we found that feces from vaginally delivered infants predicted maternal *H. pylori* status with higher accuracy than feces from C-section delivered infants (Fig. 5C). Thus, the fecal microbiota from infants is a good predictor of maternal *H. pylori *positivity, only in the case of vaginal delivery.

### Predictive functional profiling comparisons in fecal microbiota from infants by maternal *H. pylori* status according to mode of delivery

Consistent with the taxa composition results, PICRUSt analysis showed differences in predicted functional profiles by maternal *H. pylori* status in vaginally born but not in C-section delivered infants (LDA > 3.0) (Fig. 4C). Infant microbiome pathways for secretion system, metabolism functions, specifically in lipopolysaccharide biosynthesis, and ion channels pores formation were significatively increased in born to *H pylori*-positive, in comparation with *H pylori*-negative mothers. These results suggest that effects resulting from carrying *H. pylori* in the mothers, modify the functional profile of fecal microbiota in their newborns.

## Discussion

The results of this novel small observational study show that bypassing labor and natural birth exposures can lead to distal effects of maternal *H. pylori *gastric infection on their infant’s microbiome. This study is limited in the extent of the characterization of the microbiota differences. Metagenomic analyses as well as quantification of specific bacteria will greatly improve our understanding of the changes and functional implications. The results suggest a trans-maternal effect of maternal *H. pylori* -likely via the maternal microbiota or her immune status- on the bacterial transmission to and colonization of the infant. Natural exposures during labor and delivery could be important for the specific *H. pylori*-associated factors modulating the infant microbiota.

In *H. pylori* subjects, the bacterium overwhelmingly dominates the gastric microbiota, and extra-gastric sites also show differences by *H. pylori* status^1,12^. In this study we observed differencesin the fecal microbiota of *H. pylori*-positive and negative mothers only in the group of vaginal delivery, implying that *H. pylori *effects on the maternal fecal microbiota mucosal and/or systemic immune-mediated effect are enhanced by labor and birth.

We could not detect *H. pylori *antigens in the feces of newborns, unlike in the study of Merino *et al*., where they found 19.4% of 7 days old positive newborns from *H. pylori* positive mothers^14^. Other studies such as that of Kienesberger *et al*. show that of *H. pylori *status in the infant was not influenced by the mother *H. pylori* status^15^. These antecedents and our results showed that the maternal *H. pylori* infection do not have an effect over the infant *H. pylori* status at 15 days of age but exert an effect over the structure and composition of microbiota, which could be associated with a possible mucosal and/or systemic immune-mediated effect on the microbiota composition early in the newborn period of life.

The effects of the maternal microbiota on the development of the immune system in the offspring has been elegantly demonstrated in mice. Using the model of transient gestational colonization in germ-free mice, the offspring of colonized mice was found to have significant differences in the innate immune compartment, in relation to germ-free mothers never exposed to microbes^16^. Specifically, the small and large intestine had increased numbers of ILC3s cells and iMNCs cells respectively, from early life to 10 days after birth and were permanent until adulthood. Furthermore, the authors labeled *E. coli* HA107 with ^13^C and transiently colonized pregnant dams, tracking bacterial products from the maternal intestine into the mother’s milk and even into the newborn tissues^17^. These experiments demonstrate that maternal bacteria or/and transfer of microbial components or products are able to influence the immune system in the offspring, and therefore, could affect the shaping of the intestinal microbiome. Other constituents of the microbiota could be influence over the effect of *H. pylori*-induced disease. Co-infection with enterohepatic *Helicobacter* species like *Helicobacter bilis* and *Helicobacter hepaticus*, can ameliorate the proinflammatory pathology induced by the *H. pylori*-infection, with attenuated T helper1 and an active T helper 17-type cell response^18,19^.

Children infected with *H. pylori* have the unique characteristics of presenting mild gastric inflammation, lower rates of ulceration, and almost no preneoplasic lesions or gastric cancer^20^. Studies of *H. pylori *infection on childrenT cell responses show increased gastric Tregs and reduced Th1 and Th17 mediated inflammatory responses, compared with infected adults^5,6^. Case-control as well as epidemiological studies have provided evidence for an inverse relationship between *H. pylori* and allergies. In children, an epidemiologic study examining data from the US National Health and Nutrition Examination Survey (NHANES) 1999–2000 by Chen *et al*. showed an inverse association between *H. pylori *seropositivity and asthma^7^. Our group found a consistent inverse relationship between allergy markers, such as skin test positivity and total serum IgE, with *H pylori* infection in children, but not in adults^8^. The ability of inducing Tregs peripherally might not be completely mediated by *H. pylori* infection itself, but alterations in the gut microbiota early in life may have critical and long-standing effects on Treg immune responses.

Maternal *H. pylori* indirect modulation of the neonate gut microbiota may exert an effect on the development the Treg cells. This hypothesis is supportedby a recent publication of Kyburz and cols., where they demonstrated in mice that trans-maternal *H. pylori* exposure reduces allergic airway inflammation in offspring through a Treg cells mechanisms^21^. They used pregnant female mice, exposed to twice-weekly doses of *H. pylori* extracted for 3 weeks and/or during the lactation, then the offspring received a single intranasal dose of house dust mite allergen for the purpose of allergic sensitization. Posteriorly, the offspring received a pulmonary challenge with influenza A virus. The authors showed that prenatal *H. pylori* extract induced changes in the offspring microbiota, favoring gastric depletion of Firmicutes and Bacteroidetes, as well as higher frequencies of specific Treg cells subsets in the lungs. Finally they detected an enhanced demethylation of the TSDR (Regulatory T cell–specific demethylated) region within the Foxp3 locus only in Treg cells from *H pylori*–exposed but not naive mice^21^. These results suggest that maternal *H. pylori *might exert a modulatory effect in the gut microbiota, Treg profile, and epigenetic level in the newborns.

*H. pylori* colonization of the human stomach is ancestral and leads to histological mucosal evidence of immune recognition (known as asymptomatic chronic inflammation). In adults, the chronic persistence of *H. pylori* increases the risk later in life, of gastroduodenal ulceration and gastric malignancy^3^, but in early life, it has been associated with more robust immune responses, including to tuberculosis^4^. *H. pylori* exemplifies the level of complexity of our relationship with our microbiota over lifetime. The prevalence of *H. pylori*is being reduced with urbanization. In Chile prevalence is still high (73% of adults are infected), albeit the wide use of antibiotics and improvements in hygiene and global socio-economical level^22^.

Over the decades the rates of C-section deliveries have increased worldwide surpassing 30% in the US and approaching 40% in Chile^23,24^. Although life-saving in some cases, like antibiotic use, there has been overuse under the assumption of no detrimental consequences. Recent work has shown how C-section (which entitles antibiotic use) changes development and is associated with higher risk of immune and metabolic disorders in the offspring^25,26^.

A limitation of our study, a usual limitation for cohorts studies that consider follow up in home visits, is the probability of contamination with the sampling surface (for example, changing table) and its effects on the microbiota analysis. In our work the fecal samples from the infant were collected from the diaper with a sterile swab and stored in a sterile cryotube, the which was immediately frozen. Many other papers have published data based on infant fecal samples taken by parents (at home)^27–30^. In all of them, the sample collection was from the diaper, as we did in our study.

However, the risk of contamination of fecal samples at home exist, but in general, concern of contamination is smaller for samples with high bacterial load, such as feces, in relation to, for example skin. With such high bacterial load, the contaminants are negligible. In the literature, we found only one article that showed an association between the dust microbiota and infant stool communities, but their take care to place a diaper liner in the child’s diaper^31^. However, the authors did not refer to possible contamination with surface contaminants from a changing table, floor, or other such sources. Considering the previous points, we think that the more important is that all the babies were born in the Hospital (in the same hospital) and that all the samples were recollected in the same way in the home.

Other limitation of our study is the lack of functional analysis to evaluate infant microbiota with other host conditions and clinical outcomes. For this reason, a new birth cohort is under construction and follow up (ARIES cohort, registered at clinicaltrials.gov, NCT04186949) designed to evaluate how genetic and environmental factors during ‘the first 1000 days’ affect the development of allergies and asthma in children. We hope to answer relevant questions regarding functional/metabolic/immune changes in the host combining the evaluation of maternal conditions, newborn immune phenotype, microbiota, genotype, epigenetics, and environmental factors that can influence in the development of allergy and asthma in the child.

Considering our results, we could to speculate that maternal *H. pylori *modulates the composition of fecal microbiota in infants born vaginally, the which, might imply immune effects, up-regulating Treg responses which reduce risks of allergy-mediated disorders^5^. During human evolution, generations of humans were born through the birth canal of their *H. pylori*-positive mothers and changing these factors might lead to changes in the phenotype of future generations.

## Patients and Methods

### Study design and participants

In this transversal study, we recruited 22 consecutive healthy mothers and their healthy full-term neonates. Recruitment was carried out in the pre-delivery maternity area and/or nursery of the Clinical Hospital of Pontificia Universidad Católica de Chile, in Santiago. After reviewing and signing of informed consent, the participants of this birth cohort, completed an initial questionnaire. Inclusion criteria for the mothers considered healthy mothers, older than 18 years, willing to breastfeed, and living in the urban metropolitan area of the medical center. Exclusion criteria were admission to the hospital during pregnancy (other than labor and delivery), history of preeclampsia, cholestasis and other major pregnancy morbidity, and history of having received *H. pylori*-eradication therapy at any time in her life.

Inclusion criteria for the babies included: full-term newborn (delivery at a gestational age of 37–42 weeks, as determined by first day of the last menstrual period or via ultrasound dating and newborn physical evaluation) born from an eligible mother. Exclusion criteria included:admission to the hospital immediately after birth, use of antibiotics in the first 2 weeks of life, major congenital abnormalities not previously diagnosed during pregnancy.

### Stool sampling and *H. pylori* status determination

A maternal stool sample was obtained before hospital discharge. Newborns stool samples were obtained at home at 15 days of age. Stool samples were collected with a sterile cotton applicator into sterile cryotubes (for *H. pylori* detection) or in XpeditionLysis and Stabilization solution (Zymo research) for DNA extraction. Home samples were collected directly from the diaper of the newborn and stored in a −20 °C fridge to then be transferred to −80 °C freezer within 2 hours until processing.

*H. pylori* status was determined with Premier Platinum HpSA (Meridian diagnostics Bioscience, Ohio, USA) in stool samples using manufacturer instructions.

### 16S *rRNA* gene-based sequencing and analysis

DNA was extracted from the stool samples using the MoBio (CA, USA) PowerSoil®-htp 96 Well Soil DNA Isolation plates according to the manufacturer’s procedure. The V4 hypervariable region of the 16S *rRNA* gene was amplified with F515/R806 primer set and sequenced using Illumina MiSeq platform (2×150 cycles, paired-end). Sample processing, sequencing, and core amplicon data analysis were performed by the Earth Microbiome Project (www.earthmicrobiome.org), and all amplicon sequence data and metadata have been made public through the EMP data portal (qiita.microbio.me/emp)^32^.

Analyses of sequences were performed using the software package Quantitative Insights Into Microbial Ecology (QIIME, version 1.8, http://qiime.org). Sequencing reads were assigned to samples according to their barcodes. Reads with incorrect barcodes, incorrect primer sequences and/or average quality Phred score lower than 20 (Phred ≥ Q20) were removed. Chimeric reads were detected using UCHIME^33^. Operational Taxonomical Units (OTUs) were picked using UCLUST based on a distance of 0.03 (≥97% identity) with Greengenes database (v13_8). For comparison of beta diversity between communities, the unweighted/weighted UniFrac distances were used with PERMANOVA dissimilarity/similarity statistical test^34,35^. Linear discriminant analysis effect size (LEfSe) analysis was used to detect significant differences in relative abundance of bacterial taxonomy^36^. PICRUSt (phylogenetic investigation of communities by reconstruction of unobserved states) was used to predict functional metagenomics differences from the 16S *rRNA* gene dataset using KEGG orthologs classification^37,38^.

### Clinical data and questionnaires

Medical records were extensively reviewed to retrieve all the demographic and medical relevant information from the mother-newborn pair, using a preformed data sheet. In addition, a questionnaire was applied including basic demographics, mother’s medical history (including BMI, medications, GBS status, diabetes, etc.), and baby’s gestational age.

Follow up questionnaire: Records of the infant and mother weight and height, and infant´s diet were obtained at day 15. Infant feeding was classified according to current and standard criteria (ComisiónNacional de LactanciaMaterna, MINSAL, Chile) in 4 categories: exclusive breastfeeding, predominant breastfeeding, breastfeeding plus formula, and exclusive formula.

### Statistical analysis

Data validation and analysis were performed with Stata® 10.1. The chi-square test was used to compare categorical variables. The Kruscall-Wallis test was used for comparison between continuous variables. The Dunn post-test was used to make comparisons between any two groups if any statistical difference was found. Statistical significance was defined as a p value<0.05.

### Ethics

This study was approved by the Ethical Scientific Committee from the Pontificia Universidad Católica de Chile (CEC-MED UC, *Project numbers #PUC 14-077*) and all methods were performed in accordance with the relevant guidelines and regulations. The procedures and informed consent forms were explained to every mother. The written informed consent was obtained from all participants and the parents/guardians gave assent to participate in this study. Samples were handled anonymously. All sensitive clinical and demographic data were coded and recorded in an excel datasheet, which were locked with restricted access to the principal investigator in full agreement with the Good Clinical Practices and the current legal policies.

## Supplementary information

SUPPLEMENTARY MATERIALS.
