# Supplementary material for: Maternal H. pylori is associated with differential fecal microbiota in infants born by vaginal delivery
Source: Sci Rep. 2020 Apr 29;10:7305. doi: 10.1038/s41598-020-64296-7 (PMC7190710; doi:10.1038/s41598-020-64296-7)
Supplement: Supplementary file 1 — SUPPLEMENTARY MATERIALS. [file 41598_2020_64296_MOESM1_ESM.pdf]

Maternal *H. pylori* is associated with differential fecal microbiota in infants born by vaginal delivery

Caroll D. Hernandez[1&]; Hakdong Shin[2&]; Paula A. Troncoso[1#]; Macarena H. Vera[1]; Andrea A. Villagran[1]; Selena M. Rodriguez[3]; Marlene A. Ortiz[1]; Carolina A. Serrano[1]; Arturo Borzutzky[4,5]; Maria Gloria Dominguez-Bello[6*], and Paul R. Harris[1*].

[1]Department of Pediatric Gastroenterology and Nutrition, Pontificia Universidad Católica de Chile School of Medicine, Santiago, Chile

[2]Department of Food Science and Biotechnology, College of Life Science, Sejong University, Seoul, South Korea

[3]Wayne State University School of Medicine, Detroit, Michigan, USA

[4]Department of Pediatric Infectious Diseases and Immunology, Pontificia Universidad Católica de Chile School of Medicine, Santiago, Chile

[5]Millennium Institute on Immunology and Immunotherapy, Pontificia Universidad Católica de Chile School of Medicine, Santiago, Chile

[6]Department of Biochemistry and Microbiology, and Department of Anthropology Rutgers University, New Brunswick, USA

SUPPLEMENTARY MATERIALS

Figure S1. Bacterial beta diversity in infant feces by maternal *H. pylori* status according to mode of delivery. PCoA plot of bacterial communities using weighted UniFrac distances in infant born vaginally (n=13) **(A)** or C-section (n=9) **(B)**. Weighted UniFrac intragroup distances **(C)**. PERMANOVA was used to test dissimilarity. The non-parametric P values were calculated using 10,000 Monte Carlo permutations.

Figure S2. Bacterial alpha diversity in infant feces by maternal *H. pylori* status and delivery mode. The non-parametric P values were calculated using 10,000 Monte Carlo permutations.

Figure S3. *Enterobacteriaceae* and *Veillonella* taxa comparisons in intestinal microbiota from infants by maternal *H. pylori* status according to mode of delivery. Box plots showing the relative abundance of the selected bacterial taxa identified open-reference OTU picking methods based on SILVA 132 database **(A)** and EzBioCloud 16S database **(B)**.

Table S1. Sequence data from analyzed samples.

Sample source	Mother				Infant				Total
**Delivery mode**	**Vaginal**		**C-section**		**Vaginal**		**C-section**		
**Maternal *H. pylori* status**	**Negative**	**Positive**	**Negative**	**Positive**	**Negative**	**Positive**	**Negative**	**Positive**	
# of samples	6	7	5	4	6	7	5	4	44
**Total # of Seqs**	**89,632**	**280,626**	**82,554**	**83,150**	**87,699**	**285,818**	**76,177**	**89,914**	**1,075,570**
Mean # of Seqs ± SD	14,939 ±1,097	40,089 ±12,895	16,511 ±730	20,788 ±8,994	14,617 ±2,227	40,831 ±12,013	15,235 ±1,837	22,479 ±13,571	24,445 ±14,153
**Total # of Seqs yielding OTUs**	**82,896**	**259,126**	**77,074**	**75,127**	**83,571**	**267,864**	**68,614**	**84,936**	**999,208**
Mean # of Seqs yielding OTUs ± SD	13,816 ±998	37,018 ±11,935	15,415 ±628	18,782 ±7,579	13,929 ±2,016	38,266 ±11,189	13,723 ±1,176	21,234 ±12,621	22,709 ±13,129
T-test on # of Seqs yielding OTUs (p-value)	0.0011		0.4119		0.0005		0.2813		
**# of observed OTU types**	**1,169**	**1,722**	**1,184**	**1,370**	**331**	**546**	**307**	**456**	**2,845**
**Mean # of observed OTU types ± SD**	378 ±124	590 ±179	430 ±88	569 ±185	95 ±21	151 ±44	99 ±14	145 ±87	307 ±226
T-test on # of observed OTU types (p-value)	0.0457		0.2329		0.0246		0.3392		

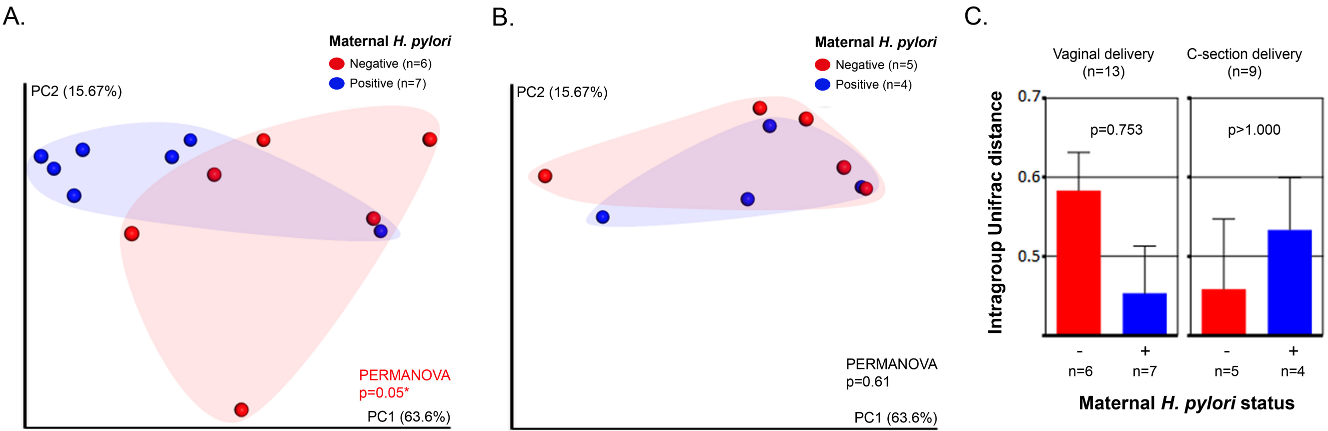

A.
Maternal H. pylori
Negative (n=6)
Positive (n=7)
PC2 (15.67%)
PERMANOVA
p=0.05*
PC1 (63.6%)
B.
Maternal H. pylori
Negative (n=5)
Positive (n=4)
PC2 (15.67%)
PERMANOVA
p=0.61
PC1 (63.6%)
C.
Vaginal delivery
(n=13)
C-section delivery
(n=9)
Intragroup Unifrac distance
0.7
0.6
0.5
p=0.753
p>1.000
-
+
n=6
n=7
n=5
n=4
Maternal H. pylori status

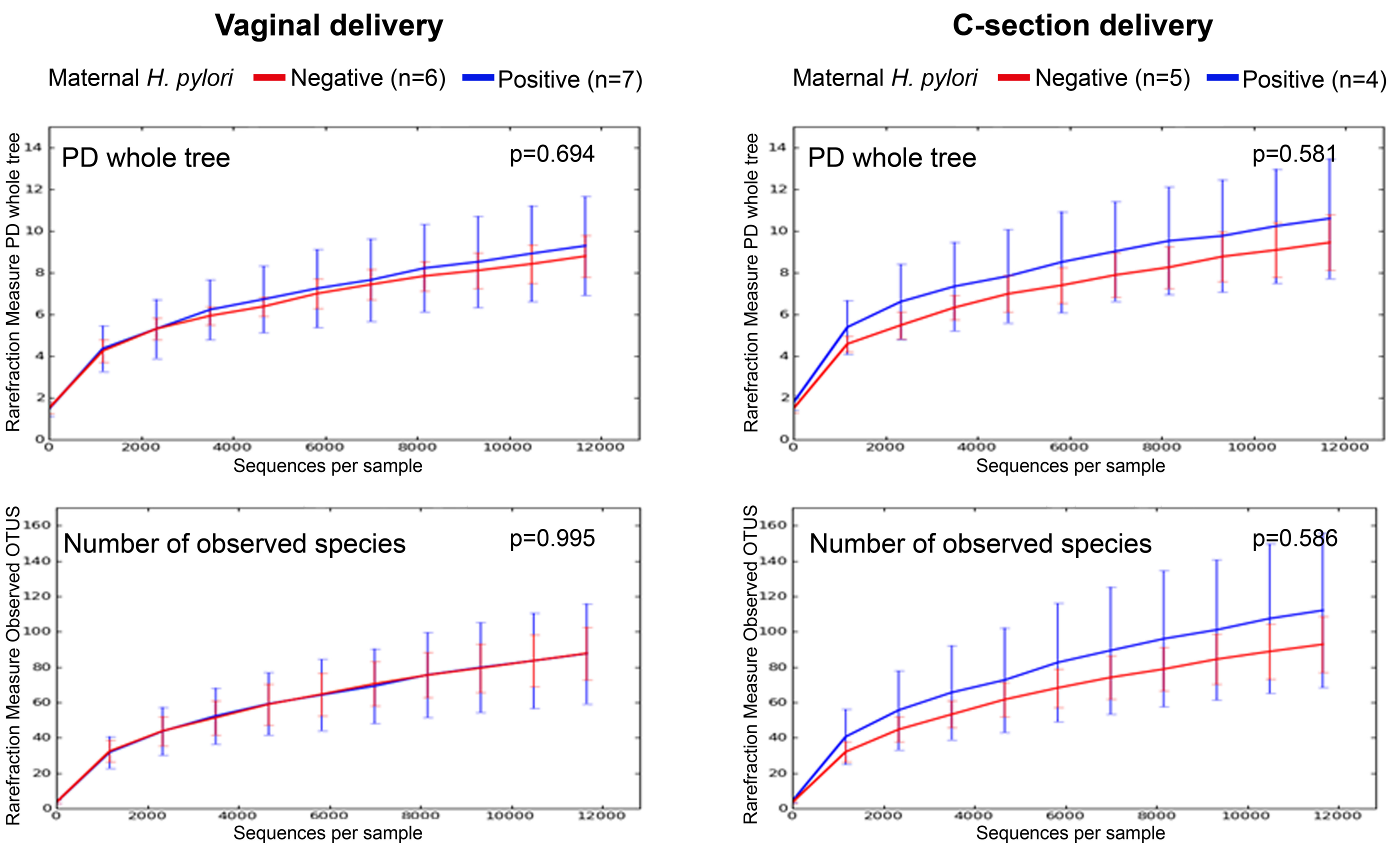
Vaginal delivery
C-section delivery
Maternal H. pylori
Negative (n=6)
Positive (n=7)
Maternal H. pylori
Negative (n=5)
Positive (n=4)
PD whole tree
p=0.694
PD whole tree
p=0.581
Rarefraction Measure PD whole tree
Sequences per sample
Number of observed species
p=0.995
Number of observed species
p=0.586
Rarefraction Measure Observed OTUS
Sequences per sample
0
2000
4000
6000
8000
10000
12000
0
2
4
6
8
10
12
14
0
20
40
60
80
100
120
140
160

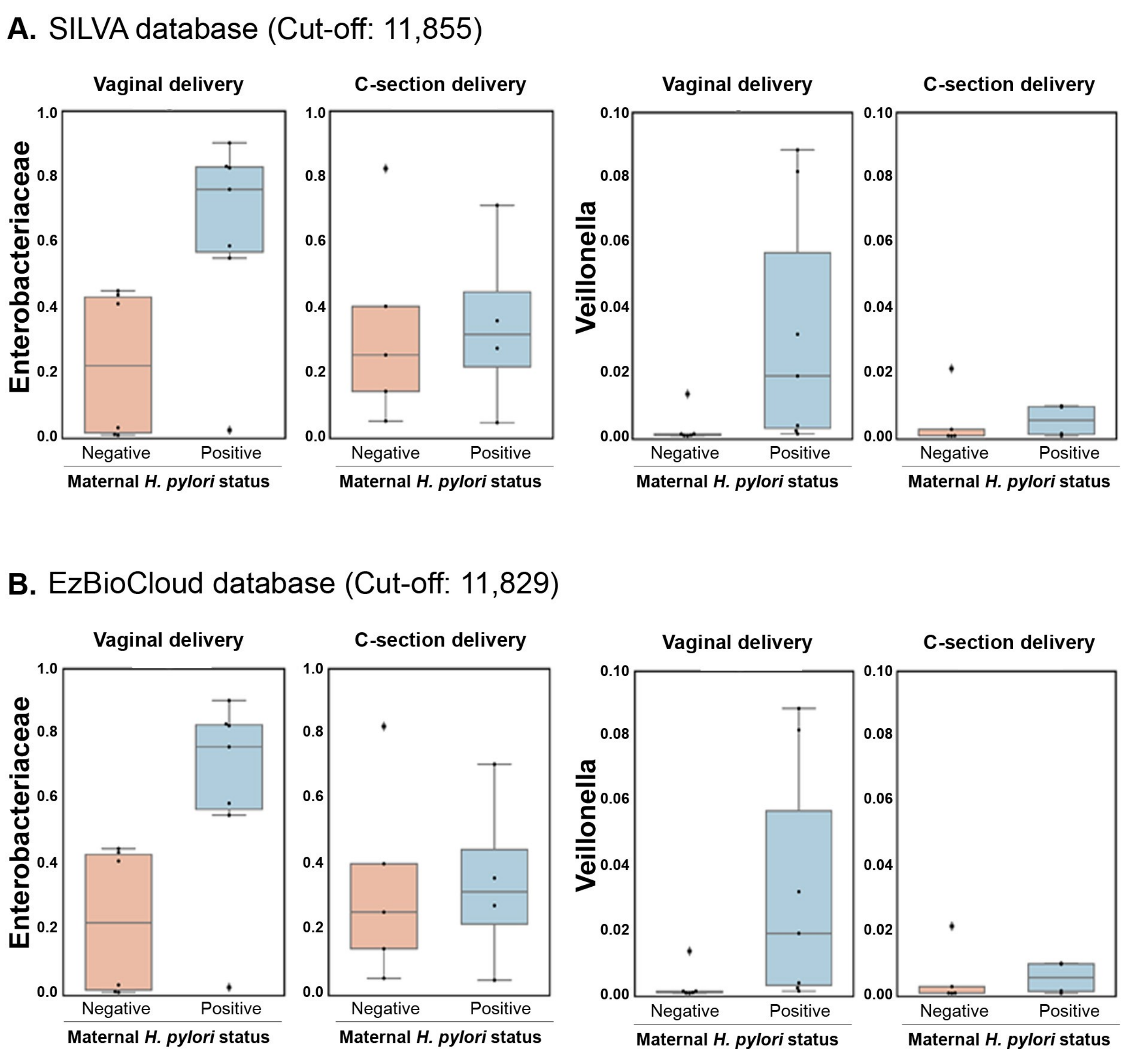
A. SILVA database (Cut-off: 11,855)
Vaginal delivery
C-section delivery
Vaginal delivery
C-section delivery
Enterobacteriaceae
Veillonella
1.0
0.8
0.6
0.4
0.2
0.0
0.10
0.08
0.06
0.04
0.02
0.00
Negative
Positive
Maternal H. pylori status
B. EzBioCloud database (Cut-off: 11,829)
Vaginal delivery
C-section delivery
Vaginal delivery
C-section delivery
Enterobacteriaceae
Veillonella
1.0
0.8
0.6
0.4
0.2
0.0
0.10
0.08
0.06
0.04
0.02
0.00
Negative
Positive
Maternal H. pylori status
